# Rare metabolic gene essentiality is a determinant of microniche adaptation in *Eschherichia coli*

**DOI:** 10.1371/journal.ppat.1013775

**Published:** 2025-12-08

**Authors:** Omid Ardalani, Patrick V. Phaneuf, Jayanth Krishnan, Siddharth M. Chauhan, David Pride, Daniel C. Zielinski, Jonathan M. Monk, Lars K. Nielsen, Bernhard O. Palsson

**Affiliations:** 1 Novo Nordisk Foundation Center for Biosustainability, Technical University of Denmark, Lyngby, Denmark; 2 Department of Bioengineering, University of California, San Diego, La Jolla, California, United States of America; 3 Department of Pathology, University of California, San Diego, La Jolla, California, United States of America; 4 Australian Institute for Bioengineering and Nanotechnology, The University of Queensland, Brisbane, Queensland, Australia; 5 Bioinformatics and Systems Biology Program, University of California, San Diego, La Jolla, California, United States of America; 6 Department of Pediatrics, University of California, San Diego, La Jolla, California, United States of America; 7 Center for Microbiome Innovation, University of California, San Diego, La Jolla, California, United States of America; University of Cambridge, UNITED KINGDOM OF GREAT BRITAIN AND NORTHERN IRELAND

## Abstract

Rare genes in bacterial pangenomes have historically been considered non-essential, dispensable, or even costly, and largely excluded from in-depth analyses due to their perceived redundancy, high variability, and presumed neutral evolutionary origin. However, whether rare genes contribute to metabolic robustness when core genes are lost remains an open question. In this study, we systematically investigate the role of rare metabolic genes in *Escherichia coli*, revealing their essentiality in maintaining metabolic functions under core gene loss. Through a pangenome-scale reconstruction of 15311 strain-specific genome-scale models (panGEM) and over 22.4 million gene knockout simulations, we demonstrate that: (i) 9.4% of rare metabolic genes are essential in at least one of three key host environments—feces, serum, and urine; (ii) 41% of strains rely on at least one rare essential metabolic gene for survival; (iii) rare metabolic genes emerge as a result of microniche adaptation, and (iv) panGEM allows for the prediction of a subset of highly conserved metabolic reactions with minimal genetic diversity as stable drug targets. These findings challenge the common view that rare genes primarily serve as evolutionary byproducts of genome fluidity and reveal their critical role in metabolic resilience.

## Author summary

Bacteria have a “pangenome”: a common set of genes shared by most strains plus thousands of uncommon, or rare, genes that appear only occasionally. Rare genes are often perceived as expendable. We asked whether some of them actually become essential when *Escherichia coli* adapts to different corners of the human body, such as the gut (feces), blood (serum), and urinary tract (urine) where nutrients and stresses differ. We built a species-wide resource of 15,311 strain-specific metabolic models (a panGEM) and ran over 22 million computer simulations that switch genes off one by one. This allowed us to test, in silico, which genes each strain truly needs to grow in each environment. Contrary to the common view, we found that rare metabolic genes frequently matter: about one in ten rare genes is essential in at least one host environment, and ~41% of strains depend on at least one such rare essential gene. These rare genes often substitute for missing “core” genes, revealing how strains tailor their metabolism to specific microniches. Together, these results recast rare genes not as genomic clutter but as a standing reserve that supports metabolic resilience and ecological fit. They also provide a framework for interpreting metabolic diversity among clinical *E. coli* isolates and for understanding why rare genes persist across strains and sites.

## Introduction

Affordable sequencing technologies led to genome data reaching petabyte scales [1,2], enabling the rise and growth of pangenomics as a new field of study [3,4]. Bacterial pangenomes exhibit remarkable genetic diversity, traditionally categorized into core genes, which are conserved across all strains, accessory genes, which contribute to many but not all strains, and rare genes, which are present in only a few strains or a single strain [5–7]. *Escherichia coli* exemplifies this diversity, possessing a large rare genome [5] shaped by horizontal gene transfer (HGT), gene fragmentation, gene loss, and selection pressures [6]. Rare genes have been widely regarded as functionally dispensable [8], often treated as transient evolutionary byproducts with little impact on core cellular processes [8]. However, recent studies have raised fundamental questions about their role in bacterial metabolism [6].

Several hypotheses attempt to explain the presence of rare genes in bacterial genomes. The complexity hypothesis [9] suggests that genes with higher connectivity are less likely to be transferred horizontally, yet our findings challenge this by showing that rare genes encode core enzymes with high metabolic connectivity. Other studies suggest that HGT-derived genes primarily confer adaptive advantages [5,10,11], that genome fluidity follows neutral evolutionary principles [12], and that rare genes often impose fitness costs and are evolutionary byproducts of high lateral gene transfer activity [13]. Moreover, some studies also regard rare genes as adaptive genes, as they may provide an evolutionary advantage under specific conditions [14]. However, we propose that rare metabolic genes are not merely adaptive or dispensable, but function as substitutes for lost core genes. This leads to an important question: Do rare genes play a vital role in bacterial metabolism, particularly in the context of core gene loss?

Here, we focus on metabolic traits at the pangenome scale. Genome-scale models (GEMs) integrate genomic data into computable frameworks that link genotypes to metabolic phenotypes [15]. Recently, GEMs have been applied to individual strains, offering insight into metabolic traits across diverse strains [16–22]. A panGEM for a species—a pangenome-scale collection of strain-specific GEMs—enables detailed systems-level studies of differential metabolic functions across the pangenome of a species [4,26,30] . These computational models act as knowledge resources, revealing both species’ core processes and strain-specific rare, unique metabolic capabilities.

PanGPRs, a compendium of gene-to-protein-to-reaction (GPR) at pangenome scale are building blocks of a panGEM that link genetic diversity to specific metabolic reactions for an entire taxon [23]. PanGPRs enhance our understanding of evolutionary relationships by connecting metabolic functions to genetic variation, showing the extent of genetic diversity supporting metabolic capabilities [6]. Our previous study on the *E. coli* panGPR revealed that rare genes are predominantly fragmented versions of core or accessory genes. In limited cases, rare genes exhibit substantial sequence divergence compared to core genes encoding the same metabolic reactions. Furthermore, panGPRs demonstrated substantial genetic diversity underlying metabolic reactions, with 26% of rare metabolic genes contributing to core metabolic functions [6].

*Escherichia coli* is a versatile bacterium capable of colonizing various sites in the human body, including the gastrointestinal tract, urinary tract, and bloodstream [24–26]. These environments differ significantly in nutrient availability, with feces, urine, and serum serving as the primary nutrient sources, respectively. Understanding how *E. coli* adapts metabolically to these diverse conditions is essential for uncovering the genetic and metabolic mechanisms underlying its survival, especially in both nutrient-rich and nutrient-limited environments [24]. Rare metabolic genes, which often encode core metabolic functions, remain underexplored regarding their contribution to metabolic plasticity and essentiality.

In this study, we used pangenome-scale knockout simulations to investigate the essentiality of rare metabolic genes of *E.coli* across media representing these key nutrient sources—feces, urine, serum—and M9 minimal media as a reference. By leveraging the panGEM, we aimed to: **(i)** determine whether rare metabolic genes are essential in specific conditions, **(ii)** assess their role in maintaining metabolic stability, and **(iii)** identify conserved metabolic reactions with minimal genetic diversity, which could serve as potential antimicrobial targets.

This work reveals that rare metabolic genes serve an essential compensatory function rather than being neutral or redundant. By identifying conserved metabolic dependencies across *E. coli* strains, we provide insights into bacterial adaptation and uncover potential metabolic vulnerabilities for therapeutic intervention.

## Results

### Expanding previous *E. coli* panGEM

**Fig 1:**
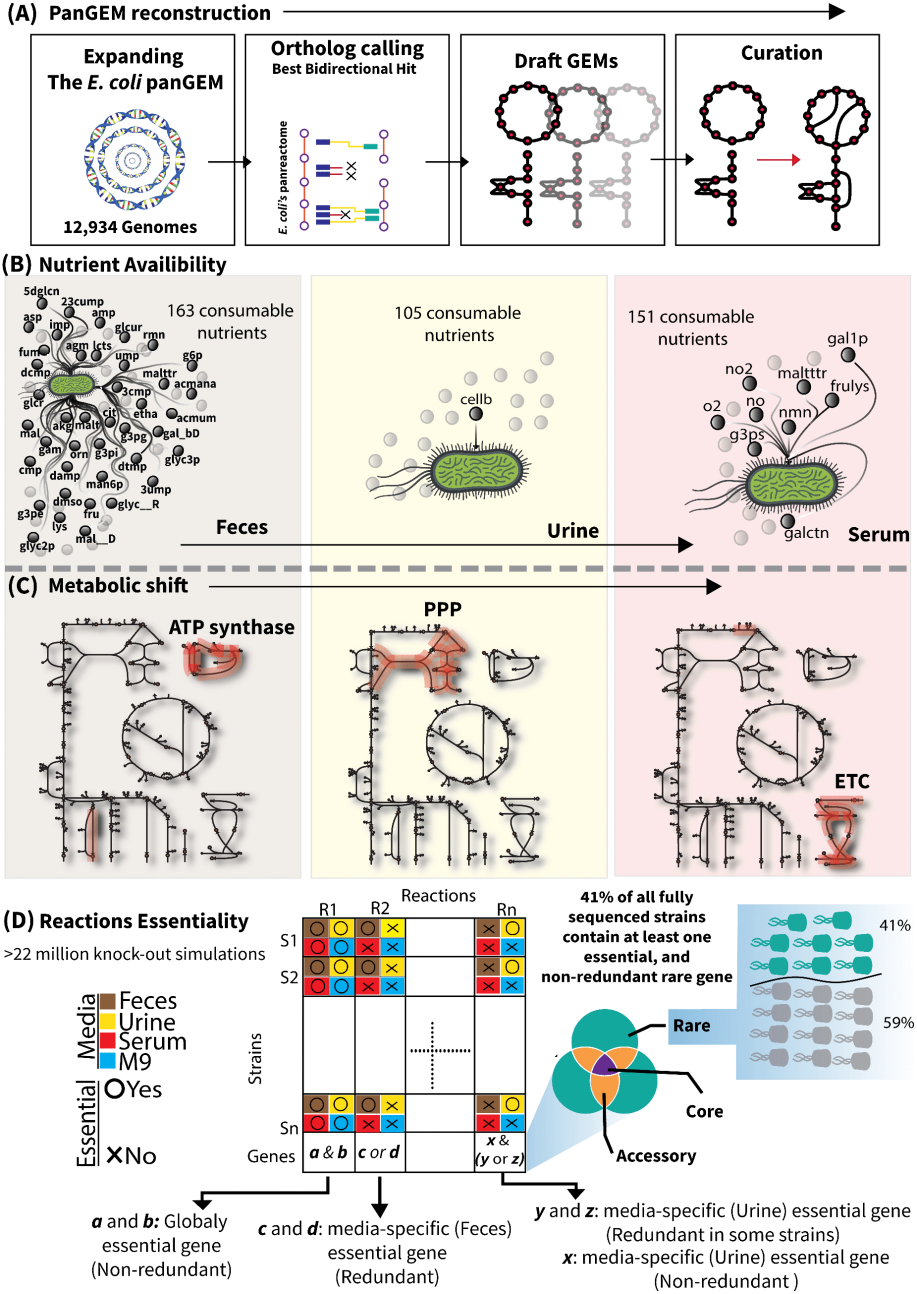
Expanding and Analyzing the *E. coli* panGEM. A) Expansion of the *E. coli* panGEM. Building on our previous work with 2,377 fully sequenced E. coli strains, we generated 12,934 new genome-scale metabolic models (GEMs) from E. coli genomes available as contigs, expanding the panGEM to encompass a total of 15,311 (2,377 previously reconstructed + 12,934 new genomes) strain-specific GEMs. These GEMs underwent semi-automated curation and gap-filling to incorporate missing gene-to-protein-to-reaction (GPR) associations. B) Nutrient availability in colonization sites. Using metabolomics data from the HMDB database [32], we simulated nutrient availability in three key colonization sites where *E. coli* is known to grow and cause infections. Flux Variability Analysis (FVA) was performed to assess the utilization potential of available nutrients in each media environment. C) Metabolic shifts across colonization sites. Parsimonious flux balance analysis (pFBA) was applied to predict metabolic shifts in uropathogenic *E. coli* (UPEC) across the simulated colonization sites, highlighting differential metabolic behaviors. D) Pangenome-scale knock-out simulations across colonization sites. Single-reaction deletion analysis was conducted across the pangenome, generating over 22 million predictions to identify essential genes.

We expanded a previously constructed, high-quality, and validated *E. coli* panGEM based on 2,377 closed genomes covering all major phylogroups [23]. Using a validated panGPR catalogue, 12,934 draft assemblies were added to create an Extended set (15,311 GEMs total) (Methods, and Fig 1A) (For detailed summaries of the genomes’ basic properties and taxonomy coverage, see S1 and S2 Figs). Two collections were thus analyzed: the Completed set (2,377 closed) and the Extended set (Completed + 12,934 draft). Bias-sensitive tasks such as panGPR reconstruction, single-gene knock-out simulations, and rare-essentiality calls were performed only on the Completed set to avoid assembly-gap artifacts (Fig 1C). Flux analyses, including FVA across media, used both sets to maximize coverage and capture metabolic diversity (Fig 1B). Comprehensive summaries and per-genome metadata are provided in S1 and S2 Figs including contigs, genome length, isolation source, country, phylogroup, mash cluster and genome status for the whole dataset), and S1 Table lists strain IDs, accessions, assembly status, lengths, contigs, isolation source/category, serotype, phylogroup, mash cluster, etc. The pangenome contains 171,199 gene clusters, of which 13,515 are metabolic (~7.9% of all clusters). Among these metabolic gene clusters, 973 are core (7.2% of metabolic), 11,866 are rare (87.8%), and 676 are accessory (5.0%). The corresponding reactome comprises 2,753 reactions, including 2,684 core (97.5%), 35 rare (1.3%), and 34 accessory (1.2%). Rare reactions were not exclusive to any strain or phylogroup; instead, they were distributed across multiple phylogroups.

The panGEM was validated by comparing model predictions, under M9 minimal medium, to published gene-knockout datasets [17,27–31] covering 850 metabolic genes across 12 strains; accuracy, precision, and F1 score were computed from confusion matrices. Model predictions were also evaluated against BioLog phenotype microarrays (96 conditions) in M9: 59 GEMs from the same collection were analyzed previously, and eight additional strains were added here, increasing BioLog validation coverage to 67 strains in total; accuracy and precision were statistically assessed. Knockout and BioLog validations were performed regardless of genome assembly status.

Subsequently, the metabolic states of strains within each environment were investigated. Second, gene essentiality across strains within all phylons were assessed, focusing on rare metabolic genes (Fig 1D). Third, the relative impacts of environmental and genetic factors on *E. coli* metabolism were compared. The term phylon is used to denote a pangenome-defined clade: a group of strains identified by non-negative matrix factorization of the gene presence/absence matrix, where each phylon represents a characteristic accessory-gene portfolio; phylons broadly match classical *E. coli* phylogroups while providing finer substructure [5].

### Distinct metabolic states of *E. coli* in urine, feces, and serum reflect environmental adaptation

The panGEM predicts metabolic states aligned with preferred nutrient availability across host environments. For pathogens to establish infection, they must adapt their metabolism to thrive within specific host microenvironments. For instance, *E. coli* transitions from a benign gut resident to a pathogen in nutrient-limited environments, such as the urinary tract. These metabolic shifts reflect the bacterium’s strategy to compete for resources while resisting host defenses. Here, we examine how *E. coli* modulates its metabolism across the microenvironments where it commonly resides or causes infection [24].

**Fig 2:**
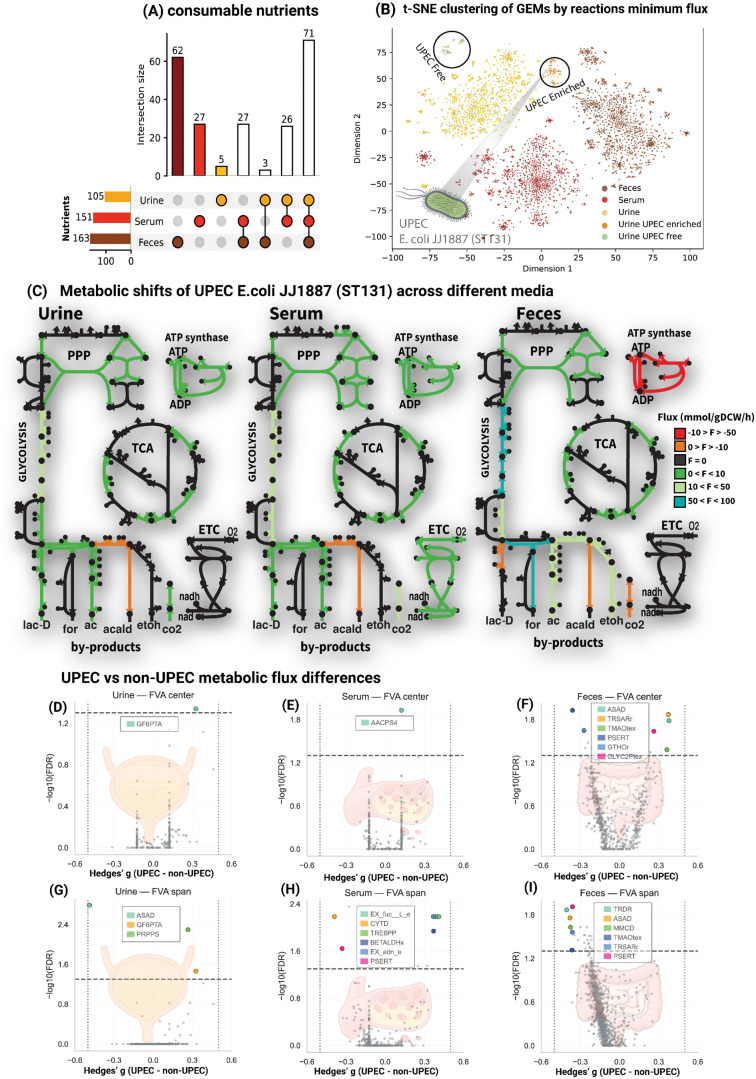
Computed metabolic states across strains in the panGEM for growth in feces, urine, and serum. (A) Overlap of consumable nutrients in feces, urine, and serum. The UpSet plot details the count of unique nutrients in each simulated condition, as well as the shared count of nutrients between pairs of conditions and among all three conditions. (B) Clustering of GEMs based on minimum reaction fluxes from FVA. The t-SNE plot illustrates the clustering of GEMs according to the minimum fluxes of their reactions during simulated growth in feces, urine, and serum. Clusters are highlighted with distinct colors corresponding to each media type (feces, serum, urine). The inset highlights UPEC-enriched and UPEC-free clusters. (C) This panel highlights the metabolic shifts that a pathogenic UPEC strain undergoes while growing in urine, feces, and serum. The metabolic maps illustrate the changes in fluxes through the TCA cycle, glycolysis, pentose phosphate pathway, ATP synthase, and the electron transfer chain, as calculated by pFBA for growth in feces, urine, and serum. The fluxes of different reactions are color-coded according to the legend. (D–I) Effect-size–aware volcano plots of reaction-level flux differences between UPEC and non-UPEC isolates across three media. Subpanels D–F show FVA center (mean of min/max flux) for urine, serum, and feces, respectively; G–I show FVA span (max–min) for the same media. Each point is a reaction (x-axis: Hedges’ g for UPEC − non-UPEC; y-axis: − log₁₀(FDR) from two-sided Mann–Whitney U with Benjamini–Hochberg correction). Positive g indicates higher flux in UPEC; negative indicates higher in non-UPEC. Grey points are non-interpretable; colored points mark the top interpretable hits within each subpanel meeting all criteria: FDR ≤ 0.05, |g| ≥ 0.5, and |Cliff’s δ| ≥ 0.33. Legends are per subpanel and list the highlighted reactions.

To capture the nutrient utilization and catabolic capacities of *E. coli* strains across three primary colonization sites—urine, feces, and serum—we conducted flux variability analysis (FVA) using the extended panGEM. This approach predicted an array of nutrients available within these environments that support *E. coli* growth. Simulated media, based on reported metabolomics data for urine, feces, and serum [32], predicted that strain-specific GEMs collectively enable *E. coli* strains to utilize 163 metabolites in feces, 151 in serum, and 105 in urine (with at least one strain of 15,000 strains utilizing each nutrient in the respective environment) (Fig 2A). Among these metabolites, 71 were shared across all three media, while the remainder were unique to each environment, including 62 exclusives to feces, 27 to serum, and 5 to urine.

FVA predicts the minimum and maximum reaction fluxes of a metabolic network under specific conditions while optimizing a defined objective function. To investigate whether the metabolic state of strains varies across feces, serum, and urine, and to contrast uropathogenic *E. coli* (UPEC) strains’ metabolism against other strains, FVA was performed. The minimum metabolic fluxes predicted for bacterial growth in these three environments were clustered using t-SNE. The analysis revealed distinct clusters highlighting the metabolic diversity among *E. coli* strains. Unlike in urine, UPEC strains in serum and feces are interspersed with non-UPEC strains and do not form discrete clusters (Fig 2B).

To define these environment-specific metabolic adaptations in greater detail, we selected a pathogenic UPEC strain (*E. coli* JJ1887, ST131) from the urine UPEC-enriched cluster. The strain represents a globally disseminated and clinically significant lineage, often associated with recurrent urinary tract infections and high virulence [33]. We then performed parsimonious flux balance analysis (pFBA) to characterize the metabolic responses of this strain within each simulated medium. The following sections provide an in-depth analysis of *E. coli* JJ1887’s metabolism in feces, serum, and urine (Fig 2C).

**Fig 3:**
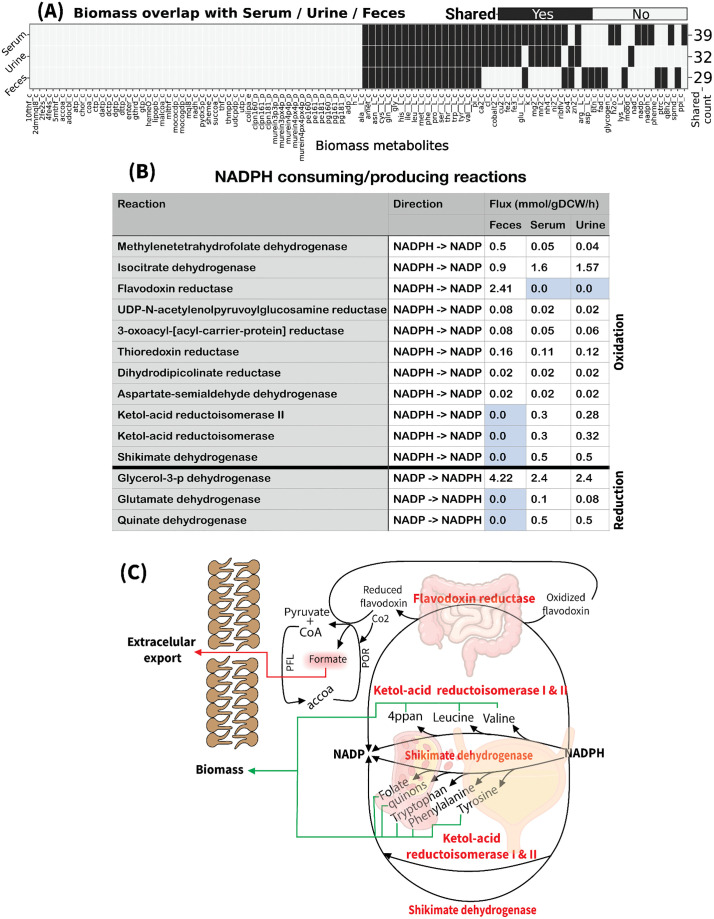
Anabolic-Catabolic Balance of the NADPH Pool in UPEC Metabolism Across Urine, Feces, and Serum. (A) Biomass–medium overlap. Binary heatmap showing the presence of biomass objective metabolites (columns) in each sample medium (rows: Serum, Urine, Feces). Columns are ordered by decreasing frequency of presence across samples to emphasize commonly shared metabolites. The right annotation column reports the total number of biomass metabolites present in each sample.(B) Predicted NADPH–producing and –consuming reactions for UPEC E. coli JJ1887 (ST131) in three media representing feces, serum, and urine. Values are parsimonious FBA fluxes (mmol gDCW ⁻ ¹ h ⁻ ¹). The “Direction” column indicates the physiological direction used for reporting: NADPH → NADP⁺ = NADPH consumption, NADP⁺ → NADPH = NADPH production. Only reactions with non-zero flux in at least one environment are shown; blue cells denote zero flux in that environment. (C) Conceptual summary of the environment-specific routing of reducing power. Reactions highlighted correspond to flux-carrying NADPH nodes from panel B, illustrating shifts in anabolic redox demand and catabolic by-products (e.g., formate) across feces, serum, and urine. Abbreviations: UPEC, uropathogenic E. coli; mmol gDCW ⁻ ¹ h ⁻ ¹, millimoles per gram dry cell weight per hour.

1) **UPEC *E. coli* JJ1887 Metabolic Adaptations in Feces: Catabolic Profile with Anaerobic End Products.** The rich availability of carbon, nitrogen, and phosphorus sources, along with accessible macromolecule precursors, drives *E. coli* to prioritize the catabolic utilization of these nutrients in feces. 29 nutrients in feces contribute directly to biomass composition (Fig 3A). The primary NADPH pool in feces is utilized predominantly by the FLDR2 (Fig 3B) reaction to reduce oxidized flavodoxin, which is subsequently oxidized by pyruvate synthase (POR), converting acetyl-CoA and CO_2_ into pyruvate and CoA, effectively regenerating NADP in a catabolic pathway rather than consuming NADPH for anabolism (Fig 3C). Additionally, high catabolic activity through carbohydrate metabolism leads to excess ATP and proton production, reversing ATP synthase activity, where protons are pumped extracellularly in exchange for ATP consumption (Fig 2C and S1 Text).
2) **UPEC *E. coli* JJ1887 Metabolic Adaptation in Serum: Anabolism-Driven Pathways Supported by Oxygen Availability.** The metabolic profile in serum shifts towards anabolism, driven by the availability of biomass precursors (39 identified) compared to feces (Fig 3A), requiring flux through anabolic pathways to synthesize essential compounds. This shift increases the activity of reactions producing and consuming NADPH (Fig 3B), steering cellular metabolism toward anabolism, in contrast to the catabolic state observed in feces. Additionally, reduced ATP and proton production from carbohydrate metabolism leads ATP synthase to import protons from the extracellular space into the cytosol, facilitating ATP generation through ADP phosphorylation (Fig 2C and S2 Text).
3) **UPEC *E. coli* JJ1887 Metabolic Profile in Urine: Anabolic Shift with Reduced Catabolic Pressure.** Urine promotes a similar anabolic state to serum, driven by the availability of biomass precursors (32 identified) (Fig 3A) and fewer carbon, nitrogen, and phosphorus sources. In contrast to feces, ATP synthase in urine supports ATP production and proton import, although at lower fluxes than observed in serum (Fig 2C). Glycolytic flux in urine is reduced relative to both feces and serum, reflecting decreased catabolic activity. Lactate (13.5 mmol/gDCW/h) emerges as the primary glycolytic end-product in urine, similar to the findings in serum. Additional end-products include acetate (0.003 mmol/gDCW/h) and CO_2_ (7.06 mmol/gDCW/h), produced at lower fluxes than in serum (Fig 2C). Similar to serum, urine exhibits a higher number of NADPH-consuming enzymes with non-zero flux directed toward anabolic pathways compared to feces (Fig 3C and S3 Text).

### Medium-specific flux signatures distinguish UPEC from non-UPEC

Reactions flux differences between UPEC and non-UPEC isolates (Fig 2D–2I) indicate medium-specific shifts in both typical flux (center) and route plasticity (span). In urine, higher center flux in UPEC was observed for glutamine-fructose-6-phosphate transaminase (GF6PTA), consistent with increased hexosamine/peptidoglycan-precursor throughput; by span, GF6PTA and phosphoribosylpyrophosphate synthetase (PRPPS) showed broader feasible ranges in UPEC, whereas aspartate-semialdehyde dehydrogenase (ASAD) displayed a negative g, indicating greater flexibility in non-UPEC. In serum, positive center effects were dominated by acyl-[acyl-carrier-protein] synthetase (AACPS4), suggesting enhanced activation of host-derived long-chain fatty acids in UPEC; span increases in UPEC were seen for L-fucose exchange (EX_fuc__L_e), trehalose phosphorylase (TREP), and adenosine exchange (EX_adn_e), while cytidine deaminase (CYTD) and phosphoserine transaminase (PSERT) carried negative g values, pointing to larger flexibility in non-UPEC. In feces, a heterogeneous pattern emerged: positive center shifts occurred for tartronate semialdehyde reductase (TRSARr), trimethylamine-N-oxide diffusion (TMAOtex), and glycerol-2-phosphate diffusion (GLYC2Ptex), whereas PSERT and glutathione oxidoreductase (GTHOr) had negative g (higher in non-UPEC). Notably, every span highlight in feces—thioredoxin reductase (TRDR), ASAD, methylmalonyl-CoA decarboxylase (MMCD), TMAOtex, TRSARr, and PSERT showed negative g, indicating systematically greater route plasticity in non-UPEC under gut-like conditions. Together, the center panels indicate directional shifts favoring UPEC in select carbohydrate, nucleotide-precursor, and lipid-activation steps, whereas the span panels indicate that non-UPEC often retains broader redox and carbon routing options, especially in feces, highlighting medium-dependent contrasts between preferred pathways and contingency capacity.

### Validation of panGEM knockout predictions and carbon source consumption

**Fig 4:**
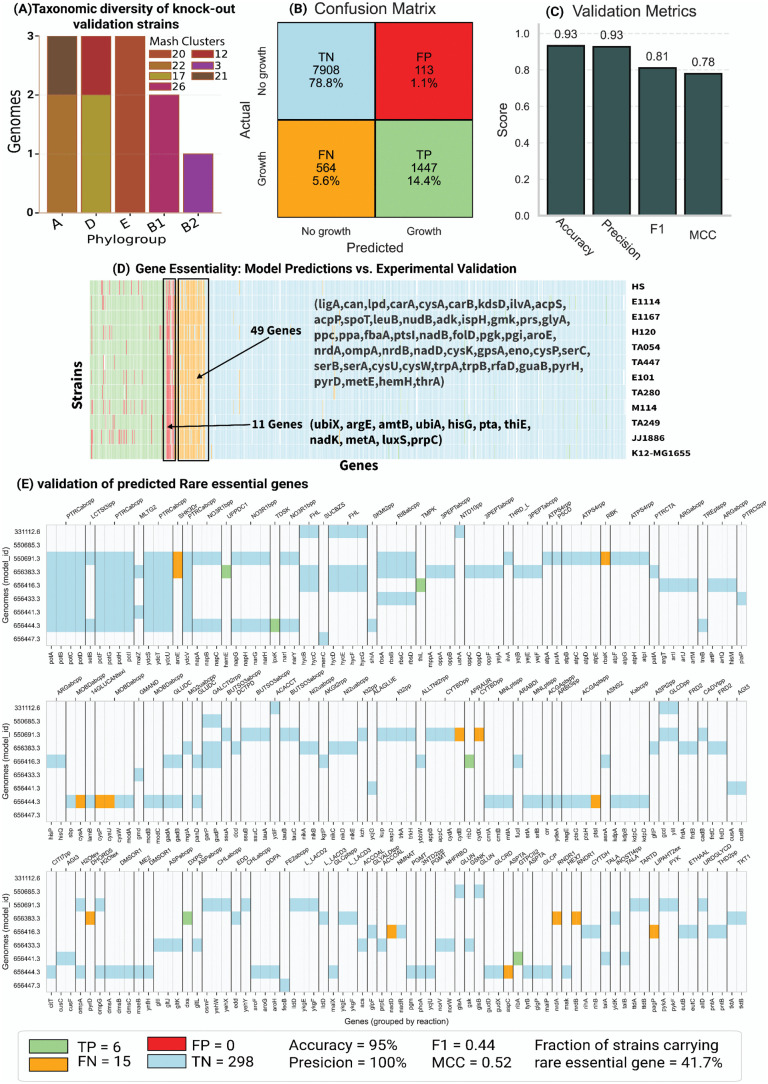
Validation of gene knock-out predictions. Knock-out predictions were evaluated against published M9 knock-out data for 850 metabolic genes across 12 E. coli strains [15–17,20,27,39]. Eleven GEMs were reconstructed from draft (contig) genomes, whereas MG1655 derives from a complete genome and shows the lowest error rate (accuracy = 93.81%, precision = 93.85%). (A) Phylogroup composition of the 12 strains used for KO validation. (B) Confusion matrix comparing model predictions (growth/no growth) with KO outcomes; cells report counts and proportions of true negatives (TN), false positives (FP), false negatives (FN), and true positives (TP). (C) Aggregate validation metrics (accuracy, precision, F1, and Matthews correlation coefficient). (D) Detailed comparison of experimental KO outcomes versus model predictions across all tests, shown as a matrix with genes as columns and strains as rows; gene names are annotated. (E) Zoomed view of rare-essential genes across genomes, grouped by the reaction they encode, showing predicted versus experimental outcomes. White cells indicate that the gene is not rare in that genome; colors denote TN (blue), TP (green), FN (orange), and FP (red). panel footer summarizes counts and derived metrics, and the fraction of strains carrying the rare-essential gene.

panGEMs were validated against experimental gene knockout data on M9 minimal media for 850 metabolic genes across 12 *E. coli* strains using available data in the literature [15–17,20,27,39]. The 12 validation strains span five major *E. coli* phylogroups—A (n = 3), D (n = 3), E (n = 3), B1 (n = 2), and B2 (n = 1)—distributed across seven Mash clusters (Fig 4A); this set includes lineages commonly associated with both enteric (A/B1/E) and extraintestinal (B2/D) backgrounds resulting in a total of 10,200 gene essentiality predictions. The predictions were categorized into True Positives (TP), True Negatives (TN), False Positives (FP), and False Negatives (FN) to assess the accuracy of the models.

Overall, most predictions aligned with the experimental data, with TN accounting for 78% of the total outcomes, followed by TP (14%), FN (5%), and FP (1%) (Fig 4B) and overall accuracy, precision, F1 score, and Matthew’s correlation coefficient (MCC) of 93%, 92.76%, 0.81 and 0.78 respectively (Fig 4C). The K-12 MG1655 GEM, reconstructed from a complete genome, demonstrated a slightly better prediction performance (93.81% accuracy and 93.85% precision) (Fig 4D), likely due to the higher quality of its genomic data compared to contig-based reconstructions for the other strains. The K-12 MG1655 GEM demonstrated marginally higher accuracy and precision in phenotype predictions compared to the gold-standard *i*ML1515 model, which reported an accuracy of 93.4% [34]. Furthermore, the collection of 12 GEMs evaluated in this study, selected based on data availability in the literature, exhibited significantly greater accuracy and precision compared to previous multi-strain *E. coli* GEM reconstructions, which achieved an accuracy of 80% [35].

Validated predictions revealed that most genes were correctly classified as either essential or non-essential across strains. However, a subset of genes, such as *ubiX*, *argE*, and *amtB*, contributed disproportionately to FP and FN predictions (Fig 4D), indicating areas for refinement of the metabolic reconstruction that the models are based on. These results highlight the robustness of the panGEM framework across a large dataset while identifying specific genes and pathways where predictive accuracy could be improved.

In the knockout validation set, 111 rare metabolic genes were found across nine strains of 12 validation strains (Fig 4E), including 298 true negatives, six true positives, 15 false negatives, and zero false positives. Performance for rare genes was 95% accuracy, 100% precision, F1 = 0.44, and MCC = 0.51. At the strain level, 5 of 12 KO strains carried at least one rare essential gene (41.7%), similar to our genome-scale prediction that ~41% of strains harbor at least one rare essential gene (Fig 6A).

**Fig 5:**
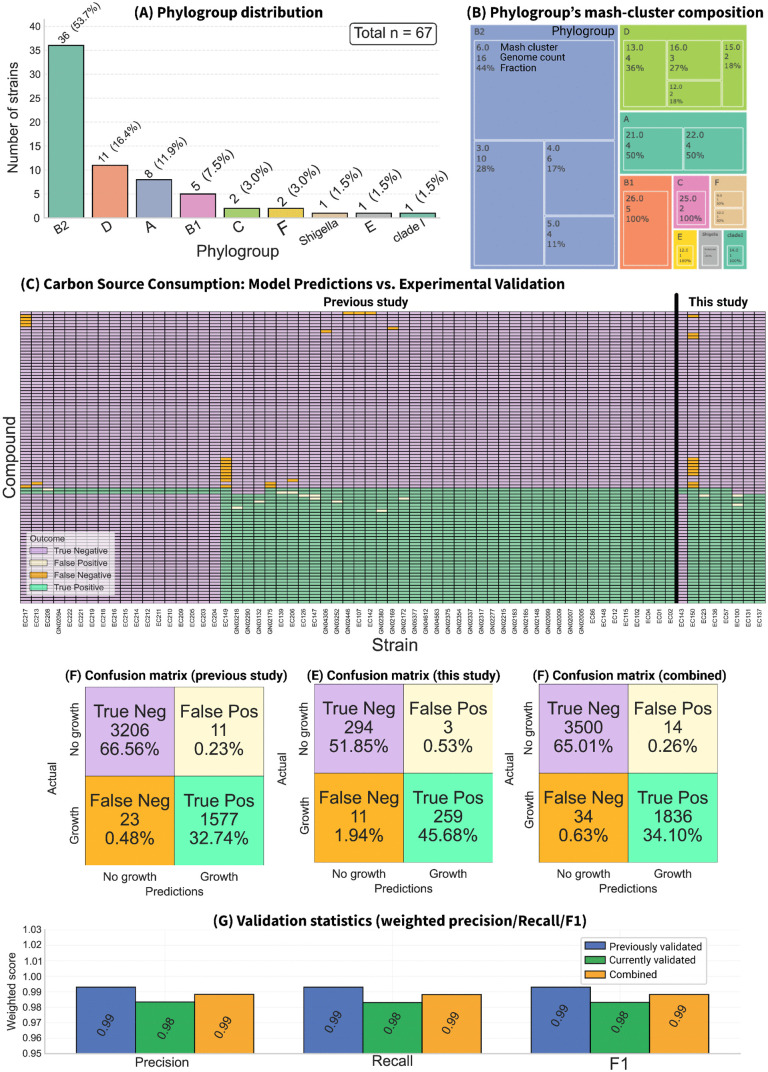
Experimental verification of panGEM predictions for carbon-source utilization. (A) Distribution of the 67 E. coli strains across phylogroups used for validation. (B) Intra-phylogroup diversity of the validation set. Annotations indicate phylogroup, Mash cluster (distance threshold 0.02), genome count, and the fraction of genomes (%) assigned to each cluster. (C) Heat map comparing model predictions with BioLog growth/no-growth outcomes across 96 carbon sources for genome-scale models (GEMs) of 67 strains (59 assessed previously and 8 newly profiled here). A version annotated with compound names is provided in S3 Fig. (D) Confusion matrix for the previously assessed strains. (E) Confusion matrix for the strains validated in this study. (F) Confusion matrix for the combined set (previous + current). Panels D–F report counts of true positives (TP), true negatives (TN), false positives (FP), and false negatives (FN) for model predictions relative to experimental outcomes. (G) Weighted performance statistics (precision, recall, and F1) for the previously assessed, newly assessed, and combined sets. Color scheme used throughout: purple, TN; orange, FN; yellow, FP; green, TP.

To further validate the panGEM a set of 67 GEMs covering all major phylogroups (Fig 5A and 5B) was validated. This set comprised 59 models from the same panGEM collection used in this study that were validated in our previous work (Omid [23]) and 8 additional models that were newly reconstructed and validated here. For each strain, predicted carbon-source utilization was compared with BioLog data across 96 compounds tested in duplicate, increasing strain-level BioLog coverage (Fig 5C). Predictions closely matched experiments, and the eight newly validated models performed similarly to the 59 previously validated models (Fig 5E vs 5D). Across all 67 models, accuracy was 99% and precision was 99% (Fig 5F). Outcome distribution was TN 62.19%, TP 36.63%, FP 0.81%, and FN 0.37% (Fig 5F).

### Plasticity in essential reactions across strains and microenvironments: identification of core and rare essential genes

Single-reaction knockouts combined with flux balance analysis (FBA) were performed across serum, feces, urine, and M9 minimal media to predict the fitness score of reactions in each strain. The fitness score was defined as the percentage reduction in growth rate. Reactions with a fitness score exceeding 95 were classified as essential. This approach yielded a comprehensive map of reaction and gene essentiality across strains and simulated conditions. Across >22.4 million simulated knockout phenotypes, most reactions and their genes were non-essential. Of the 2,753 reactions, 234 (8.5%) were essential and 2,519 (91.5%) were non-essential. Similarly, of 13,515 metabolic gene clusters, 1,370 (10.1%) were essential and 12,145 (89.9%) were non-essential.

**Fig 6:**
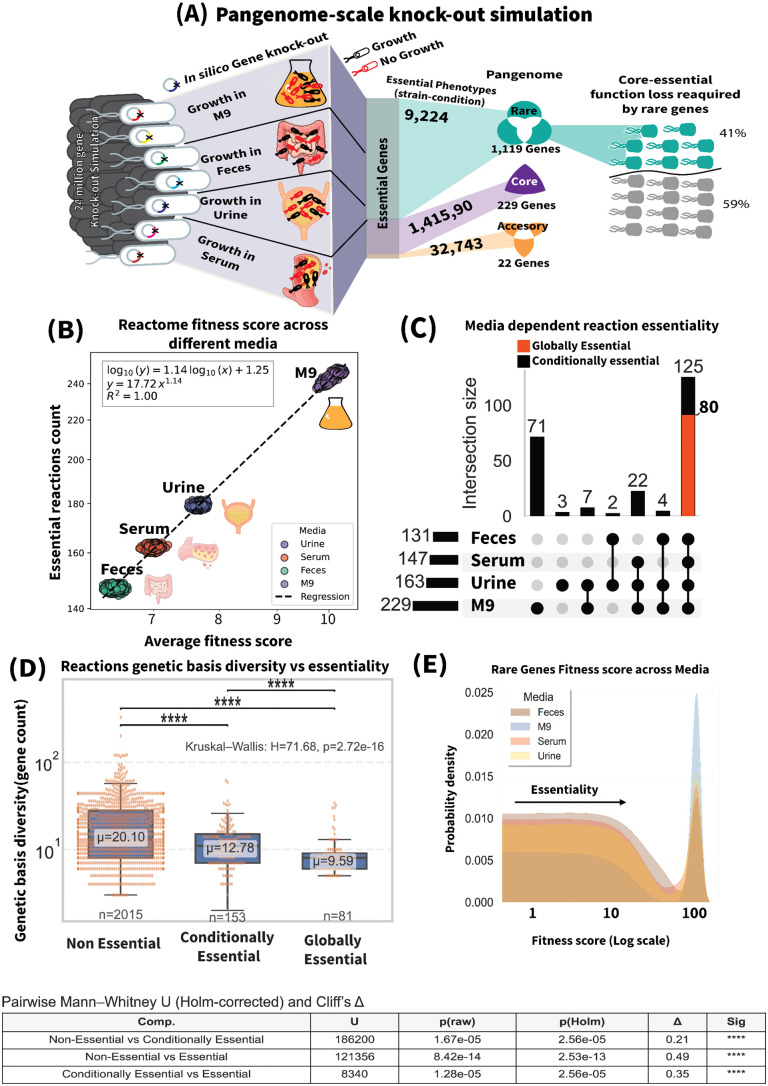
Pangenome scale knock-out simulation across different media, and the identification of globally essential metabolic reactions . (A) Single reaction deletion analysis across 2377 strains in four different media resulted in 22.4 million knock-out phenotypes, revealing essential genes of E. coli, these essential genes are grouped based on their pangenome category, Illustrating how the distinct genes in each category contribute to the number of essential phenotypes. (B) Average fitness scores of strain-specific metabolic networks across different media. Each dot represents a strain’s metabolic network, with the Y-axis indicating the count of essential reactions (fitness >95%) per strain and the X-axis showing the average fitness score of each strain’s metabolic network. Annotations highlight the media in which fitness scores were predicted. (C) UpSet plot illustrating the number of media-dependent essential reactions and their overlaps across simulated media. Reactions that are essential across all media and consistent across all strains, referred to as globally essential reactions, are highlighted in red. (D) Reactions Genetic Basis Diversity vs Essentiality: Each box represents an essentiality category (Essential, Conditionally Essential, and Non-Essential). Dots indicate individual reactions within each category. The Y-axis displays the genetic basis diversity. Other statistics are presented in the legend table. (E) Fitness Scores of Rare Genes Across Media: The density plot illustrates the distribution of fitness scores for rare essential genes, color-coded by simulated media. The X-axis represents fitness scores (0 = non-essential, 100 = essential).

Mapping reactions to the panGPRs identified 1,119 rare (linked to 3,553 no growth phenotypes), 229 core (linked to 397,877 no growth phenotypes), and 22 accessory essential genes (linked to 15,138 no growth phenotypes) across all four simulated media. Screening for these rare essential genes revealed that 41% of strains in the panGEM had at least one essential reaction coded solely by rare genes (Fig 6A).

Expectedly, the average fitness score of strain-specific metabolic networks and the count of essential reactions both decrease as media complexity increases. Minimal media, such as M9, exhibit the highest average fitness scores and the highest number of essential reactions, followed by urine, serum, and feces in descending order (Fig 6B). This variation corresponds to 229 distinct essential reactions in M9, 163 in urine, 147 in serum, and 131 in feces (Figs 6C and S4). Out of 234 essential reactions across all media, only 80 (45.8%) were consistently essential (S2 Table) hereafter referred to as globally essential reactions, 154 reactions were classified as conditionally essential, meaning they are inconsistently essential either across strains, media, or both. The remaining 2,015 reactions were called non-essential reactions. This result indicates that while *E. coli* transits from a nutrient rich environment (feces) to a nutrient limited environment (urine, serum, and M9) the selection pressure on its metabolic pathways increases. Therefore, reactions that are not essential in feces become conditionally essential while moving to nutrient limited media.

### Nutrient availability drives genetic basis diversity of metabolic reactions

Previous studies using panGPRs have demonstrated the genetic diversity of metabolic reactions across *E. coli* [23]. This diversity arises primarily from rare genes, which are often fragmented versions of core or accessory genes or acquired through horizontal gene transfer. We hypothesized that core genes may become non-essential in nutrient-rich environments (S5 Fig), allowing for a relaxed selection that facilitates genetic diversity by enabling gene loss, acquisition, and disruption. However, selection pressure eliminates unfit variants when the organism transitions to nutrient-limited environments.

Based on the hypothesis that nutrient availability influences selection pressure and thereby genetic diversity, we predicted that non-essential reactions would exhibit higher genetic diversity, as they are less constrained by selection. In contrast, under higher selection pressure, conditionally essential reactions would have a more limited diversity confined to modified genes that remain functional. Globally essential reactions were expected to show the lowest diversity, as any non-viable genetic changes would not persist.

To test this hypothesis, we analyzed the genetic diversity of reactions in three groups: non-essential reactions, conditionally essential reactions, and globally essential reactions. The results showed that non-essential reactions had significantly higher diversity (average gene count: 20.10; maximum gene diversity: 328), while globally essential reactions exhibited the lowest diversity (average gene count: 9.59; maximum gene diversity: 34). Conditionally essential reactions fell in between (average gene count: 12.68; maximum gene diversity: 62) (Fig 6D).

A Kruskal-Wallis test revealed significant differences in genetic basis diversity among the three groups (*test statistics (*H) = 71.68, p < 0.001). Pairwise Mann-Whitney U tests confirmed these differences: essential versus non-essential reactions (U = 41859.0, corrected p < 0.001), essential versus conditionally-essential reactions (U = 4053.5, corrected p < 0.001), and non-essential versus conditionally-essential reactions (U = 186200.5, corrected p < 0.001). These findings align with our hypothesis.

Given that the diversity of a reaction’s genetic basis is influenced by the presence of rare genes across the pangenome, we also examined the occurrence density of rare essential genes in four simulated environments: M9, urine, serum, and feces. The results showed significantly higher densities of rare essential genes in M9, a nutrient-poor medium, compared to the nutrient-rich environments of feces, serum, and urine (Fig 6E). Among these, feces exhibited the lowest density of rare essential genes, reflecting its enriched nutrient status. This suggests that rare genes acquired under relaxed selection in nutrient-rich environments become essential and are retained under nutrient-limited conditions.

### Globally essential reactions with minimal genetic variability as potential key targets for drug development

Mapping genes associated with media-independent globally essential reactions to the pangenome revealed considerable gene variability within the panGEM. S-adenosylhomocysteine nucleosidase (AHCYSNS), Dimethylallyltranstransferase (DMATT), Thiamine-phosphate diphosphorylase (TMPPP), Riboflavin synthase (RBFSa), 3,4-Dihydroxy-2-butanone-4-phosphate synthase (DB4PS), UDP-3-O-(3-hydroxymyristoyl)glucosamine acyltransferase (U23GAAT), Geranyltranstransferase (GRTT), 5’-deoxyadenosine nuclosidase (5DOAN) had the fewest distinct genes (five), while certain transport reactions, such as CA2tex, CLtex, COBALT2tex, and CU2tex, were each supported by up to 42 distinct genes. Many of these were rare genes, with only one or two core genes associated with each reaction.

Some globally essential reactions, such as MEPCT and MECDPS in the methylerythritol phosphate pathway, highlight the compensatory role of rare genes. MEPCT is encoded by one core gene in 2,128 strains and six rare genes in 39 strains spread across 15 phylons. Similarly, MECDPS is encoded by one core gene in 2,288 strains and six rare genes in 17 strains spread across 9 phylons (full list of globally essential reactions in S1 Table and S4 Text).

### Distribution of rare-essential genes by pathway

**Fig 7:**
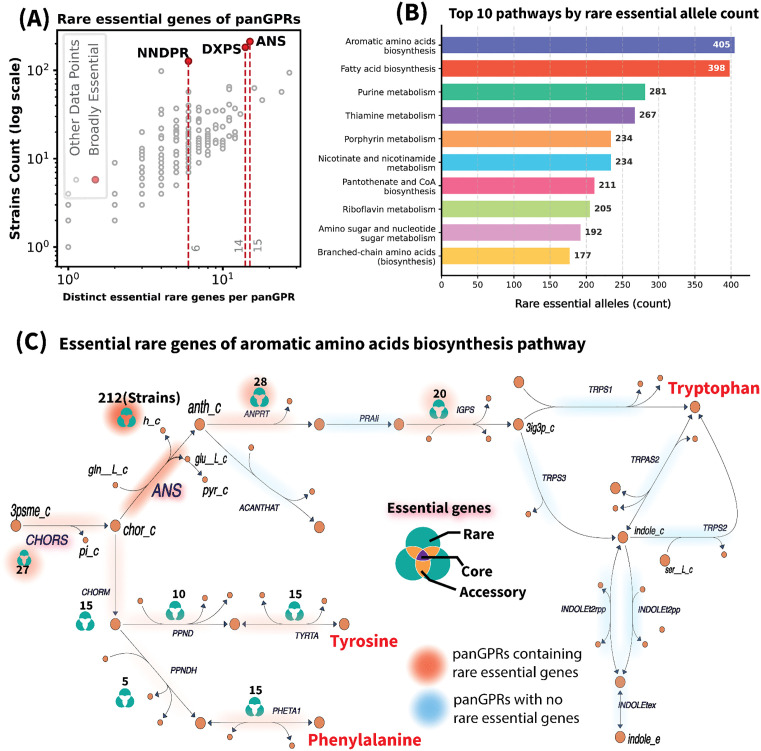
Most Frequent Essential Reactions Encoded by Rare Genes. (A) The scatter plot shows the number of distinct rare essential genes versus the count of strains in which they are present. The highlighted data points represent reactions encoded by widely distributed rare essential genes, specifically Anthranilate synthase (ANS), 1-deoxy-D-xylulose 5-phosphate synthase (DXPS), and Nicotinate-nucleotide diphosphorylase (NNDPR). (B) Top 10 pathways by count of rare-essential alleles. Bars show the absolute number of rare essential alleles mapped to each pathway (annotated at bar ends) and are ordered from highest to lowest. (C) The metabolic map illustrates the biosynthesis pathway of L-tryptophan. Anthranilate synthase (ANS), the reaction most frequently coded by rare essential genes, is highlighted to illustrate its diverse genetic basis by mapping its panGPR against the pangenome. The categories “Core,” “Rare,” and “Accessory” indicate the number of strains coding for the ANS reaction by each gene category. The red shading in the metabolic pathway highlights reactions encoded by rare essential genes across strains. The intensity of the shading reflects the prevalence of these rare essential genes in encoding the respective reactions across strains. The blue shading highlights reactions that contain no rare essential genes, indicating that these reactions are non-essential.

The genetic basis of essential reactions mapped to the pangenome showed 196 reactions associated with rare genes in various strains (Fig 7A). Anthranilate synthase (ANS), 1-deoxy-D-xylulose 5-phosphate synthase (DXPS), and Nicotinate-nucleotide diphosphorylase (NNDPR) were identified as reactions with the highest prevalence of rare essential genes across species (Fig 7A). Pathway analysis revealed that rare essential genes were particularly abundant in the aromatic amino acid biosynthesis pathway. Other pathways with high count of rare essential genes were fatty acid biosynthesis, purine metabolism, thiamine metabolism, nicotinate and nicotinamide metabolism, porphyrin metabolism, pantothenate and CoA biosynthesis, riboflavin metabolism, amino sugar metabolism, and branched-chain amino acids biosynthesis (Fig 7B). In aromatic amino acid biosynthesis pathways, rare genes of the ANS alone are essential in 212 strains (Fig 7C). Notably, one of these rare genes encoded ANS in 135 strains, the highest prevalence for a rare gene in this pathway. These findings reveal specific reactions within pathways that could be hypothesized as experiencing elevated selection pressure, as demonstrated by the prevalence of rare essential genes compensating for core metabolic functions. The presence of multiple rare variants fulfilling essential reactions indicates that these enzymes may be critical adaptation points, where genetic diversity is favored to meet specific metabolic demands. Such prevalence of rare strain-specific genes in conditionally essential reactions indicates ongoing selective forces, potentially driven by environmental pressures or niche specialization within the host.

## Discussion

panGEMs are knowledge bases that detail the metabolism and genetic composition of taxonomic groups [36–40]. These enriched knowledge bases allow for phenotype prediction at the pangenomic scale, offering insights into genetic and phenotypic diversity. panGPRs map metabolic genes to metabolic reactions and thus are the building blocks of panGEMs, containing all possible genetic variants that can drive a particular metabolic reaction within a given taxon [6].

In our previous study, we formulated a species-level panGPRs for *E. coli*. By analyzing these panGPRs, we demonstrated that the genetic basis of each reaction is diverse [23]. We found that this diversity is primarily driven by the presence of rare metabolic genes, with 94% of these rare genes coding for core metabolic reactions [6]. Further analysis revealed that rare metabolic genes have diverse genomic neighborhoods. In contrast, core and accessory genes have conserved genomic neighborhoods [6].

In the current study, we expanded our previous panGEM from 2,377 strain-specific GEMs to a larger set of 15,311 strain-specific GEMs. It covers a large number of genomes from all the major phylogroups of *E. coli,* thus representing the pangenome of *E. coli* as a species. We assessed the essentiality of reactions in this set of strains and their associated genes through pangenome-scale knockout simulations. These simulations covered more than 22.4 million knockout phenotypes across three key colonization sites—feces, urine, and serum—as well as M9 minimal media, offering the most comprehensive set of metabolic phenotype predictions to date.

The discovery that 41% of the analyzed strains rely on rare genes for essential metabolic functions is particularly revealing. This finding challenges the current hypothesis that rare genes are typically non-essential and only occasionally beneficial for adaptability. Instead, it highlights the dynamic nature of bacterial evolution, where rare genes play a crucial role in the metabolism of bacteria.

Previously, we uncovered an extensive diversity in the genetic basis of many metabolic reactions across the *E. coli* pangenome [23], revealing an interesting duality: while core functions are consistently preserved, their genetic basis is remarkably fluid. This robust genetic diversity ensures the resilience of core functions across the pangenome. Rare genes, as predicted with the panGEM of this study, often serve as an essential gene reservoir of metabolic functions, providing flexibility and adaptability in response to genetic perturbations at the population level.

Among 80 globally essential reactions, riboflavin synthase (RBFSb) and a few other targets emerge as promising antibiotic targets. RBFSb exhibits a low gene diversity, with only six genes coding for it across the pangenome—one core gene present in 2,183 strains and five rare genes found in just eight strains. This combination of robust essentiality and low genetic diversity makes RBFSb an ideal candidate for antibiotic development, a prospect already supported by previous studies [41] and shown to be essential in Loria Bertani (LB) [28] enriched, LB-Lennox [27] and LB broth [29] media in three independent studies. Other promising targets include MECDPS and MEPCT, two key reactions of the terpenoid biosynthesis pathway, which are encoded by seven distinct genes each, and exhibit relatively low genetic diversity, highlighting their potential as antibiotic drug targets. These reactions were previously shown to be essential in the LB-enriched [28], LB-Lennox [27] and LB broth [29] media.

Leveraging available metabolomics data, panGEM also predicts nutrient consumption profiles for the entire *E. coli* species in three key colonization sites: feces, urine, and serum. These simulations effectively model the metabolism of planktonic *E. coli* as it transitions between the gastrointestinal tract, urinary tract, and bloodstream, offering a dynamic view of the bacterium’s metabolic states across different body sites. This capability provides valuable insights into the mechanisms underlying various *E. coli* infections, such as uropathogenesis. Notably, t-SNE clustering of metabolic fluxes across panGEM during growth in urine successfully differentiated a UPEC-enriched cluster, highlighting the tool’s potential to distinguish between pathogenic strains.

The metabolomics data used to simulate feces, serum, and urine were based on aggregated samples from multiple individuals, rather than personalized data, even though the nutrient composition of these sites can vary significantly between individuals. This variability suggests an exciting opportunity to harness panGEM for personalized diagnostics, monitoring, and treatment. By quantitatively estimating metabolic shifts and nutrient availability, panGEM could become a powerful tool in personalized medicine. For instance, the model predicts that cellobiose could serve as a carbon source for *E. coli* growth in urine despite being authorized by European Commission as a novel food allowing it to be added to variety of food and food supplements (ELI: http://data.europa.eu/eli/reg_impl/2023/943/oj); therefore, dietary modifications that reduce cellobiose intake might reduce the growth potential of *E. coli* in urinary tract infections, offering a novel strategy for infection control. This study is limited to *E. coli*; generalizability to other taxa remains unexplored. Essentiality calls depend on the biomass objective/formulation, and alternative biomass definitions may shift reaction- and gene-level essentiality assignments. Future work spanning additional bacterial taxa will test the generality of these patterns.

## Methods

### 1- Genome selection and annotation criteria

We previously constructed an *E. coli* panGEM of 2,377 strain-specific GEMs [23]. In the present work we expanded this resource by generating 12,934 additional strain-specific GEMs and merging them with the prior set, yielding a panGEM comprising 15,311 GEMs. Beginning in 2021, we downloaded the full BV-BRC [42] genome metadata and removed all non-*Escherichia coli* records as well as entries consisting only of plasmid sequences. We imposed no restriction on isolation source; genomes from any source were eligible provided they met quality criteria. “Complete” assemblies were retained only when L50 equaled 1 and N50 exceeded 4,000,000. Draft (fragmented) assemblies were first limited to ≤355 contigs and then screened with CheckM; only genomes with estimated contamination <3.1% and completeness >98.1% were kept. The same workflow was applied to *Shigella* strains and, analogously, to *E. coli* assemblies retrieved from NCBI RefSeq. All retained assemblies were deduplicated and merged, subjected to additional redundancy/quality screening (e.g., Mash-based filtering), and uniformly re-annotated with PROKKA (V1.14.5) [43] to harmonize gene calls across sources. For pangenome analyses requiring a high-quality gene presence/absence matrix we generally restricted the dataset to complete assemblies to minimize fragmentation bias; however, we made a single exception for the 12 strains used in our gene essentiality validation experiments. When complete assemblies were unavailable for these validation strains, their corresponding fragmented assemblies were included so that their essential genes could be mapped within the pangenome and classified as core, accessory, or rare.

### 2- Pangenome reconstruction

Genomes were collated into a pangenome by clustering genes on the basis of their nucleotide sequences using CD-HIT (V4.8.1) (word size *-n* 5, minimum identity *-T* 80%, minimum alignment length *-aL* 80%, with all other parameters set to default). CD-HIT’s greedy, non-transitive clustering avoids chain-merging of remote homologs and therefore retains low-abundance gene families as separate clusters when similarity falls below the chosen cutoff. This design decision reflects our study goal of maximizing sensitivity to rare genes while keeping clustering rules explicit and reproducible. This process generated gene clusters representing allelic families across the pangenome, with all alleles within a cluster sharing at least 80% nucleotide identity. Paralogous genes were identified as clusters in which a single genome contributed more than one allele. The presence of each gene cluster was coded as “1” and its absence as “0,” yielding a binary presence–absence (PA) matrix with gene clusters as rows and genomes as columns. The PA matrix was then analyzed to classify clusters into frequency categories: core, accessory, and rare.

Core, accessory, and rare fractions of the pangenome were determined using a cumulative gene distribution approach as described previously [5]. In brief, the cumulative distribution of gene clusters forms an S-shaped curve characterized by an inflection point. The core genome was defined by identifying the upper endpoint of the curve and selecting clusters located within 90% of the distance between the inflection point and this endpoint, which corresponds to the curve’s “elbow.” Conversely, the rare genome was defined using the lower endpoint of the curve in relation to the inflection point, while the remaining clusters between these extremes were considered accessory genes. Using this method, the thresholds were: core >96.7%, accessory 6.8–96.7%, and rare <6.8%.

### 3- Panreactome and panGEM reconstruction

We first assembled an *E. coli* panreactome that served as the template for all strain reconstructions. Enterobacteriaceae models were downloaded from the BiGG database (V1.6) (http://bigg.ucsd.edu/) and all gene–protein–reaction (GPR) rules were extracted. To ensure a consistent reference, reaction, and metabolite identifiers were normalized across models before combining them into a single nonredundant set of GPRs. When mapping selected genomes to this curated GPR collection, we identified 67,054 strain-specific genes (alleles) that lacked a BiGG assignment; these were queried against KEGG (V110.0) (genome.jp/kegg/pathway.html), yielding 30 reactions not present in any of the BiGG models. Incorporating these newly formulated reactions with the existing set produced a catalogue of 2,753 metabolic reactions. Each reaction was then linked to one or more gene clusters, defining the panGPRs. The resulting panreactome comprised 13,515 metabolic gene clusters underpinning 2,753 reactions and was subsequently used as the reference for ortholog calls during panGEM reconstruction.

Strain-specific GEMs were then built by projecting this panreactome onto each re-annotated target genome using our previously developed pipeline [36]. Panreactome gene sequences and reaction/GPR information were compared to each genome by bidirectional BLAST (BLAST+2.16.0), and orthologs were called at a 90% similarity and 80% coverage threshold to map genome genes to panreactome GPRs. The initial draft GEM for each strain yielded a list of locus tags, which we cross-checked against the corresponding genome annotations to identify metabolic genes missing from the model. Missing genes were re-queried against the BiGG and KEGG databases to recover reaction annotations; genes mapping to reactions already present in a model were appended to the relevant GPRs, whereas genes associated with novel reactions were curated and added by manual reaction formulation following established protocol [44]. The biomass objective function from iML1515 [27] was incorporated into all strain models. GEMs corresponding to our in-house validation strains then underwent gap filling on M9 medium using our previous method [4], after which flux balance analysis on M9 was used to confirm biomass production.

### 4- PanGEM validation

#### 4-1- Gene essentiality validation.

To validate the models’ predictions regarding gene essentiality, we utilized gene knock-out libraries for 12 *E. coli* strains tested on M9 minimal media, as reported in the literature [15–17,20,27,39] *.* The corresponding GEMs from our pangenome-scale analysis were employed to predict the essentiality of 850 metabolic genes identified in these studies. Flux balance analysis (FBA) was performed on each of the 12 GEMs to simulate the effects of gene knockouts. The medium was set to M9, and biomass production was used as the objective function. For each simulation, the upper and lower bounds of each reaction were constrained to zero to represent a reaction knock-out. This process was repeated for all reactions in each model. A reaction was considered essential if the predicted biomass production fell below a threshold of 0.001. The genetic architecture underlying each essential reaction was then analyzed to determine gene essentiality. Reactions encoded by a single gene or by a set of genes encoding subunits of a protein complex were classified as having essential genes. In contrast, reactions with gene copies or isozymes were considered non-essential. Subsequently confusion matrix was generated using the scikit-learn library to summarize true positives, false positives, true negatives, and false negatives. Additionally, accuracy and precision scores were calculated to quantify the predictive performance of the models.

#### 4-2- Carbon source utilization using Biolog PM assays.

Eight strains from the in-house collection were profiled on Biolog phenotype microarray plates PM01 and PM02 with M9 as the base medium. Precultures were grown in M9 supplemented with 4 g/L glucose at 37 °C with shaking. Prior to inoculation, cells were washed to remove carryover carbon sources. Plates were loaded following the manufacturer’s guidance and run on an OmniLog instrument for 48 h at 37 °C. Each strain was assayed in two biological replicates.

Signal processing and feature extraction. Time-resolved respiration signals for each well were smoothed using a Savitzky–Golay filter. From the smoothed curves, the following features were extracted per well: (i) maximum respiration value, (ii) peak respiration rate, (iii) time to maximum respiration, and (iv) area under the curve (AUC).

Control-based thresholds and statistical testing. For plates containing a negative control, distributions of maximum respiration values from control wells were used to define background thresholds. Experimental wells were compared against these control distributions via statistical tests (z-tests or t-tests, as appropriate) to determine significant metabolic activity. Plates exhibiting significant control signals were flagged for manual inspection to identify contamination or other anomalies. After remediation, re-analysis was performed and valid samples reinstated.

Replicate-level calling rules. Substrate utilization was assigned per strain–compound pair using replicate consistency: 1 = utilized (most replicates positive), 0 = not utilized (replicates negative), and 0.5 = uncertain (inconclusive replicates). Strain–compound pairs with inconsistent growth/no-growth calls across replicates were excluded from the high-confidence evaluation set.

In silico comparison and performance metrics. Computational growth/no-growth predictions were generated under media definitions matched to the Biolog conditions. A growth-rate threshold of 0.001 h ⁻ ¹ was applied to distinguish no-growth outcomes. Agreement between model predictions and Biolog calls was summarized using a confusion matrix (true positives, true negatives, false positives, false negatives), from which accuracy and precision were computed. Only Biolog assays with concordant biological replicates (both growth or both no-growth) were retained; assays with discordant replicates were excluded from evaluation. Assays involving dipeptides (e.g., Ala–Gly) were also excluded because the requisite aminopeptidase steps are represented as orphan reactions (no gene associations) in the panGEM, which could otherwise inflate true-positive calls.

### 5- Mapping GPRs to pangenome and reconstruction of panGPR

Genes of panGEM GPRs were mapped to our reconstructed pangenome by their locustags to identify gene cluster of each locustag, subsequently all gene clusters coding for the same reaction were identified and grouped based on their reactions to reconstruct panGPR which shows genetic basis of same reaction across pangenome of *E. coli.*

### 6- Media simulations

M9 minimal media was obtained from previous reports [17] and for colonization sites including feces, serum and urine, their media were formulated using metabolomics information obtained from The Human Metabolome Database (HMDB) [32], for this metabolite information of feces, serum and urine was obtained and mapped to BiGG database, to find their corresponding metabolite names using their InChI Key, HMDB ID, KEGG ID, Biocyc ID and if none were found in BiGG we searched the metabolite name and corresponding synonymes, subsequently exchange reactions for found metabolites were anonymously set to -0.5 mmol/gDCW/h to allow models uptake nutrients in a rate limited way. During all constraint based analysis in this paper, maximized biomass production was the objective function of models. See S3-S5 Tables for detailed information about formulated media.

### 7- Flux variability analysis (FVA)

All simulations were performed in COBRApy v0.27.0 using the GLPK v5 linear solver. For each GEM and for each formulated medium representing feces, serum, and urine, exchange reaction bounds were set to match the corresponding in silico media definitions (see Media formulation). The biomass reaction was used as the objective, and growth was maximized as the objective function. FVA was computed with COBRApy’s “cobra.flux_analysis.flux_variability_analysis” to obtain minimum and maximum fluxes for all reactions under the specified medium. Unless stated otherwise, defaults were used: fraction_of_optimum = 1.0 (FVA at the optimal growth level) and loopless mode disabled. To accommodate the large cohort size, a per-model time limit of 60 seconds was enforced; any instance exceeding this limit was skipped and logged as a timeout. Models that were infeasible under a given medium (i.e., no feasible growth-optimal solution) were retained in the analysis framework with NA FVA bounds for that medium and were not retried under alternative settings. For each successfully solved instance, the full vector of per-reaction [min, max] flux bounds were recorded for downstream comparative analyses across media and model sets.

### 8- GEMs clustering based on minimum flux of their reaction while simulated growing in urine, feces and serum

The minimum fluxes for all reactions were determined using Flux Variability Analysis (FVA) for each media, resulting in a comprehensive dataset of flux values corresponding to each strain-media combination. Subsequently, the t-SNE algorithm [45] was applied to this dataset to reduce its dimensionality and facilitate visualization of the GEMs’ clustering behavior. The t-SNE was configured with two components for dimensionality reduction, a fixed random state to ensure reproducibility, and tailored settings for early exaggeration to optimize cluster resolution. The resulting two-dimensional embedding provided a clear representation of the metabolic similarities and differences among the GEMs across the various media. To explore potential enrichment of Uropathogenic *Escherichia coli* (UPEC) within specific clusters, we integrated strain metadata from the BV-BRC database, which includes information on pathogenicity. This metadata was used to annotate the GEMs, allowing for the identification of clusters potentially enriched with UPEC strains. The analysis identified distinct clusters associated with the different media, with particular attention given to those that exhibited a higher concentration of UPEC-related strains.

### 9- Identification of *E. coli* JJ1887 UPEC metabolic shift while growing in urine, feces and serum

Parsimonious Flux Balance Analysis (pFBA) was performed to predict flux distribution of *E. coli* JJ1887, a Fatal UPEC strain, in human urine, feces with following parameters, fraction_of_optimum = 1 and objective function = biomass. The obtained flux values were used to illustrate the bacterium core metabolism using Escher [46].

### 10- UPEC vs non-UPEC Differential flux analysis

We performed a reaction-wise, two-group comparison of FVA outputs to identify flux differences between UPEC and non-UPEC isolates. This section builds directly on the FVA procedure described above (Section 7). It uses the same per-strain FVA minimum/maximum solutions obtained for urine, serum, and feces (no additional simulations were run here). For each reaction and strain, we computed two quantities from FVA bounds: (i) center = (min + max)/2 and (ii) span = (max − min). For each medium we formed two matrices (center and span; reactions × strains). Strains were labeled UPEC or non-UPEC using BV-BRC metadata. For every reaction we compared UPEC vs non-UPEC using a two-sided Mann–Whitney U test. P-values were adjusted across reactions by Benjamini–Hochberg FDR control. In parallel we computed two effect sizes: Hedges’ g (small-sample–corrected standardized difference of means) and Cliff’s δ (rank-based effect; directionally consistent with g). Reactions were deemed interpretable only if they simultaneously satisfied FDR ≤ 0.05, |g| ≥ 0.5 (medium effect), and |δ| ≥ 0.33 (medium effect). Reactions not meeting all three thresholds were plotted in grey.

### 11- Reaction fitness score prediction

Genes were classified as essential based on specific criteria. A gene is considered essential if it encodes an essential reaction (fitness > 95%) and is the sole gene responsible for that reaction in the genome. In cases where a gene-protein-reaction (GPR) association involves multiple genes, the classification depends on their functional relationships. If the genes are isozymes or can independently encode the essential reaction, none of the genes are considered individually essential. However, if the genes encode subunits of a protein complex that is required for the reaction, each subunit is deemed essential, making all associated genes essential as well. In all cases, we did not consider essential reactions that were added to their models during the gapfilling step.

### 12- Statistical analysis of genetic variability across essential, non-essential and conditionally essential reactions

To analyze the genetic variability of metabolic reactions, reactions were categorized into three groups: globally essential, conditionally essential, and non-essential. Genetic diversity was defined as the number of distinct genes encoding each reaction across the pangenome. The normality of genetic diversity distributions within each group was assessed using the Shapiro-Wilk test. Homogeneity of variances was evaluated with Levene’s test. Based on the outcomes of these preliminary tests, non-parametric methods were applied, as the data did not meet the assumptions for parametric testing. The Kruskal-Wallis test was used to compare genetic diversity across the three reaction groups. Pairwise comparisons between groups were conducted using the Mann-Whitney U test. To account for multiple comparisons, Bonferroni correction was applied to adjust p-values. All analyses were conducted using Python. The scipy library was employed for statistical testing, while multiple comparison corrections were implemented using the statsmodels package. The complete workflow was documented, and scripts are available on https://zenodo.org/records/14853235,

### 13- Clustering genomes based on pairwise mash distance

Pairwise Mash (V2.3) distances were computed for all downloaded genomes and used as the basis for clustering. Genomes were first partitioned into six taxonomic bins—Escherichia coli, Shigella sonnei, Shigella boydii, Shigella dysenteriae, Shigella flexneri, and other Shigella spp.—and, within each bin, genomes exceeding the 99th percentile of Mash distance from that bin’s reference strain were removed to exclude outliers. Following [47] Mash distances were transformed to Pearson correlation coefficients and then to correlation distances for downstream clustering. We conducted a sensitivity analysis over candidate cutoffs in hierarchical clustering implemented via seaborn’s clustermap, selecting 0.1 as the threshold that preserved representation of all major E. coli phylogroups.

## Supporting information

S1 Fig Overview of the E. coli extended (complete + incomplete) genome set and metadata. (DOCX)

S2 Fig Overview of the E. coli completed genome set and metadata. (DOCX)

S3 Fig Carbon source consumption validation.
(DOCX)

S4 Fig Essential reactions across different media. (DOCX)

S5 Fig Urine-Specific Essential Reactions. (DOCX)

S1 Table Genomes Metadata. (XLSX)

S2 Table Predicted globally essential reactions across panGEM compared to E. coli K-12 MG1665 essentiality in Loria Bertani (LB) media. (XLSX)

S3 Table Feces media formulation. (XLSX)

S4 Table Urine media formulation. (XLSX)

S5 Table Serum media formulation. (XLSX)

S1 Text Feces Environment-Specific Uptake Profile. (DOCX)

S2 Text Serum Environment-Specific Uptake Profile. (DOCX)

S3 Text Urine Environment-Specific Uptake Profile. (DOCX)

S4 Text Media-Specific Reaction Essentiality. (DOCX)
