# Supplementary material for: Rare metabolic gene essentiality is a determinant of microniche adaptation in Eschherichia coli
Source: PLoS Pathog. 2025 Dec 8;21(12):e1013775. doi: 10.1371/journal.ppat.1013775 (PMC12704874; doi:10.1371/journal.ppat.1013775)
Supplement: S1 Fig — (DOCX) [file ppat.1013775.s001.docx]

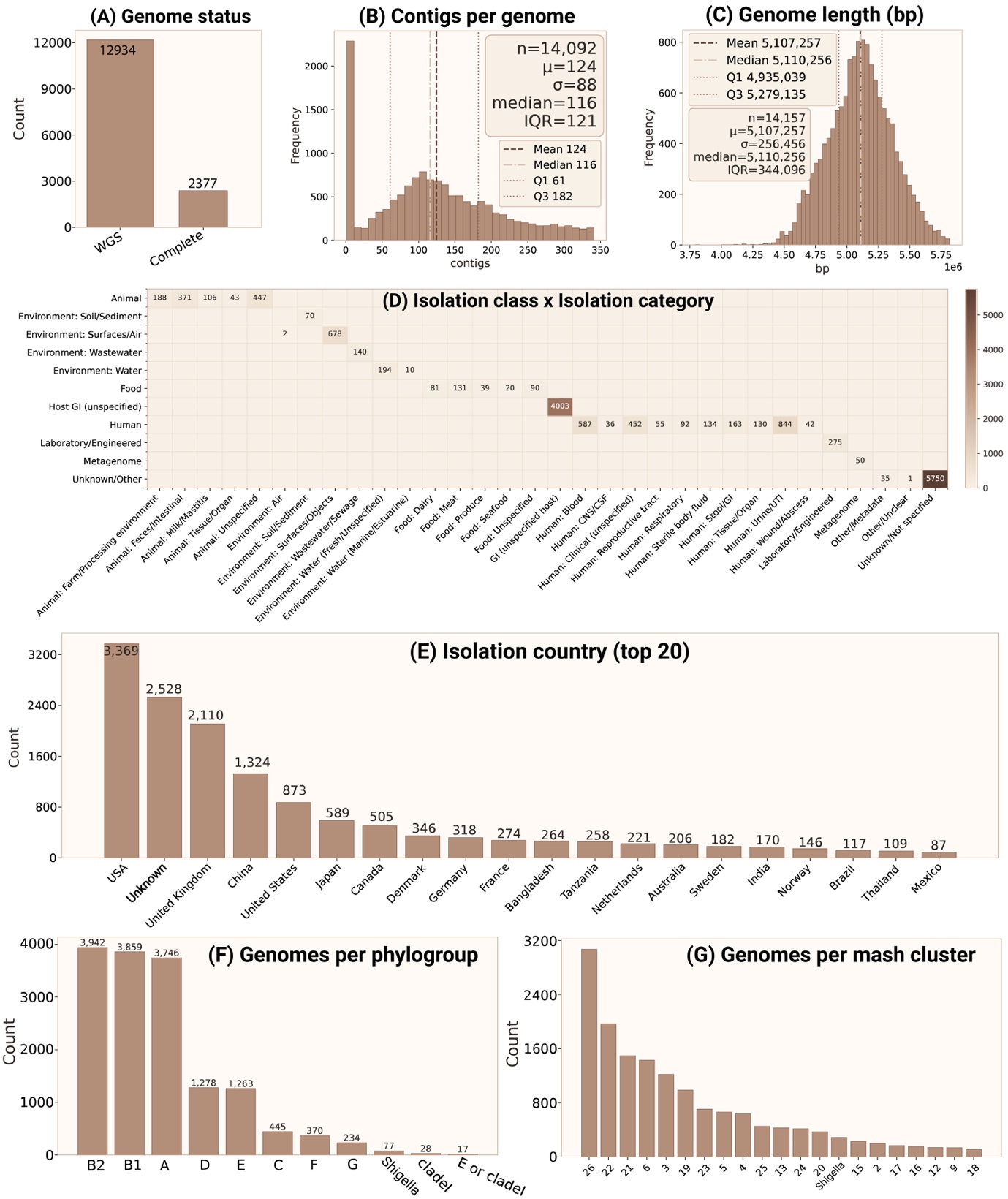


***S1 Fig. Overview of the* E. coli *extended (complete + incomplete) genome set and metadata.*** *(A) Genome status. Count of assemblies labeled as draft whole-genome shotgun (WGS) and complete genomes.(B) Assembly fragmentation. Distribution of contig counts per genome (histogram). Summary statistics (n, mean, median, SD, IQR and quartiles) are shown in the inset; vertical dashed lines mark mean and quartiles.(C) Genome length. Distribution of total assembly length (bp) across genomes (histogram) with summary statistics (n, mean, median, SD, IQR and quartiles) in the inset; vertical dashed lines mark mean and quartiles.(D) Isolation context. Heatmap of sample isolation class (y-axis) by isolation category (x-axis). Cell annotations show counts; the color bar indicates intensity (higher = more genomes).(E) Geography. Bar chart of the top 20 isolation countries by number of genomes (left-to-right in descending order); “Unknown” reflects records without a country.(F) Phylogroups. Counts of genomes assigned to major E. coli phylogroups (A, B1, B2, C, D, E, F, G, Shigella Clade I).(G) Genetic clustering. Distribution of genomes per Mash cluster (bar chart), summarizing how assemblies partition by sequence similarity.*
