# Supplementary material for: Rare metabolic gene essentiality is a determinant of microniche adaptation in Eschherichia coli
Source: PLoS Pathog. 2025 Dec 8;21(12):e1013775. doi: 10.1371/journal.ppat.1013775 (PMC12704874; doi:10.1371/journal.ppat.1013775)
Supplement: S3 Fig — Carbon source consumption validation. (DOCX) [file ppat.1013775.s003.docx]

***
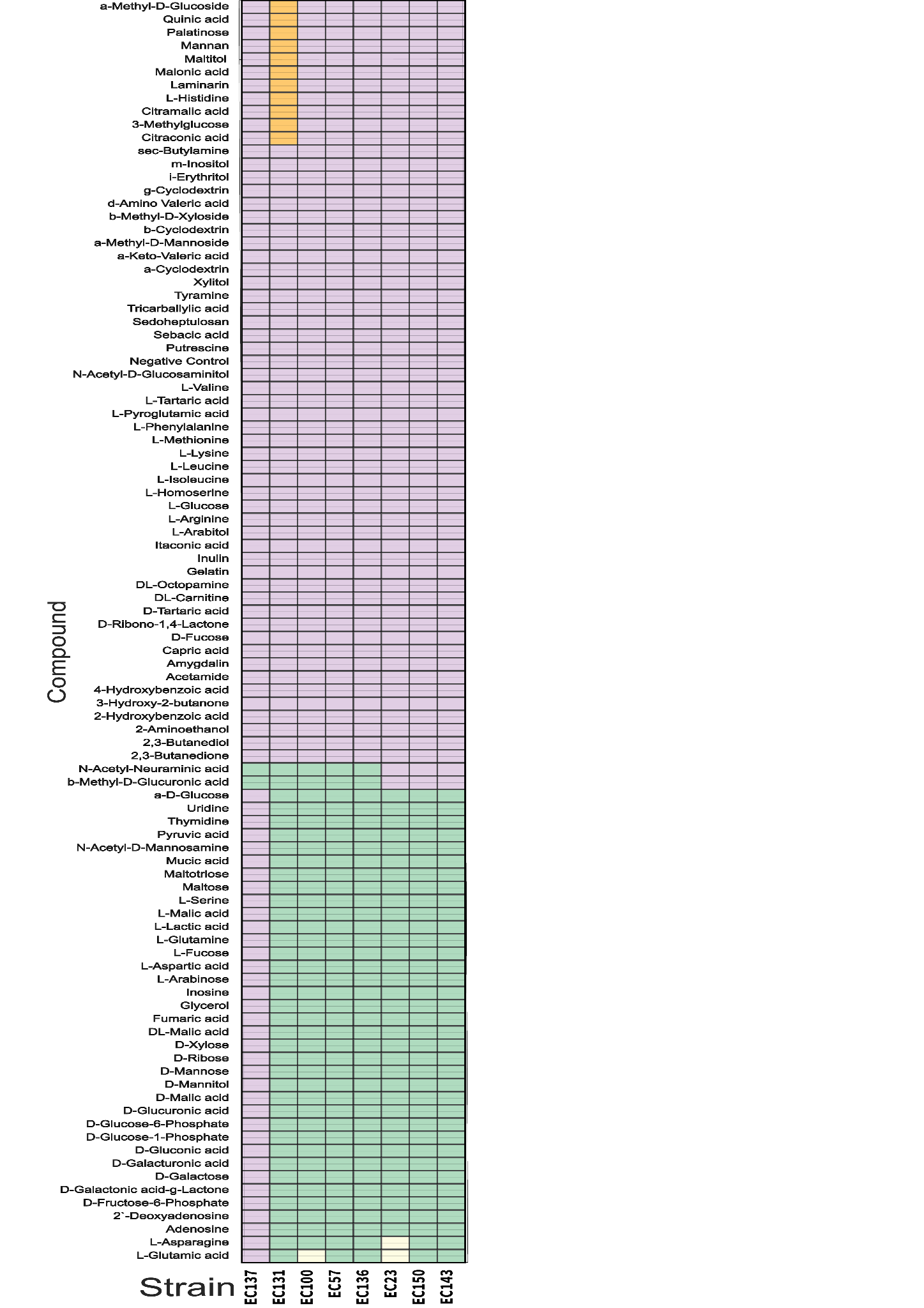
***

***S3 Fig.*** *Heatmap displaying the validation results of carbon source consumption predictions for GEMs of eight in-house sequenced strains compared to BioLog experimental data. Compounds are grouped by their respective categories (e.g., amino acids, carboxylic acids) and ordered within each group by their predominant prediction category. The first column highlighted with red borders is negative control (M9 media without carbon source).Color coding (blue: TN, orange: FN, red: FP, green: TP) is consistent across all panels.*
