# Supplementary material for: Rare metabolic gene essentiality is a determinant of microniche adaptation in Eschherichia coli
Source: PLoS Pathog. 2025 Dec 8;21(12):e1013775. doi: 10.1371/journal.ppat.1013775 (PMC12704874; doi:10.1371/journal.ppat.1013775)
Supplement: S4 Fig — (DOCX) [file ppat.1013775.s004.docx]

***
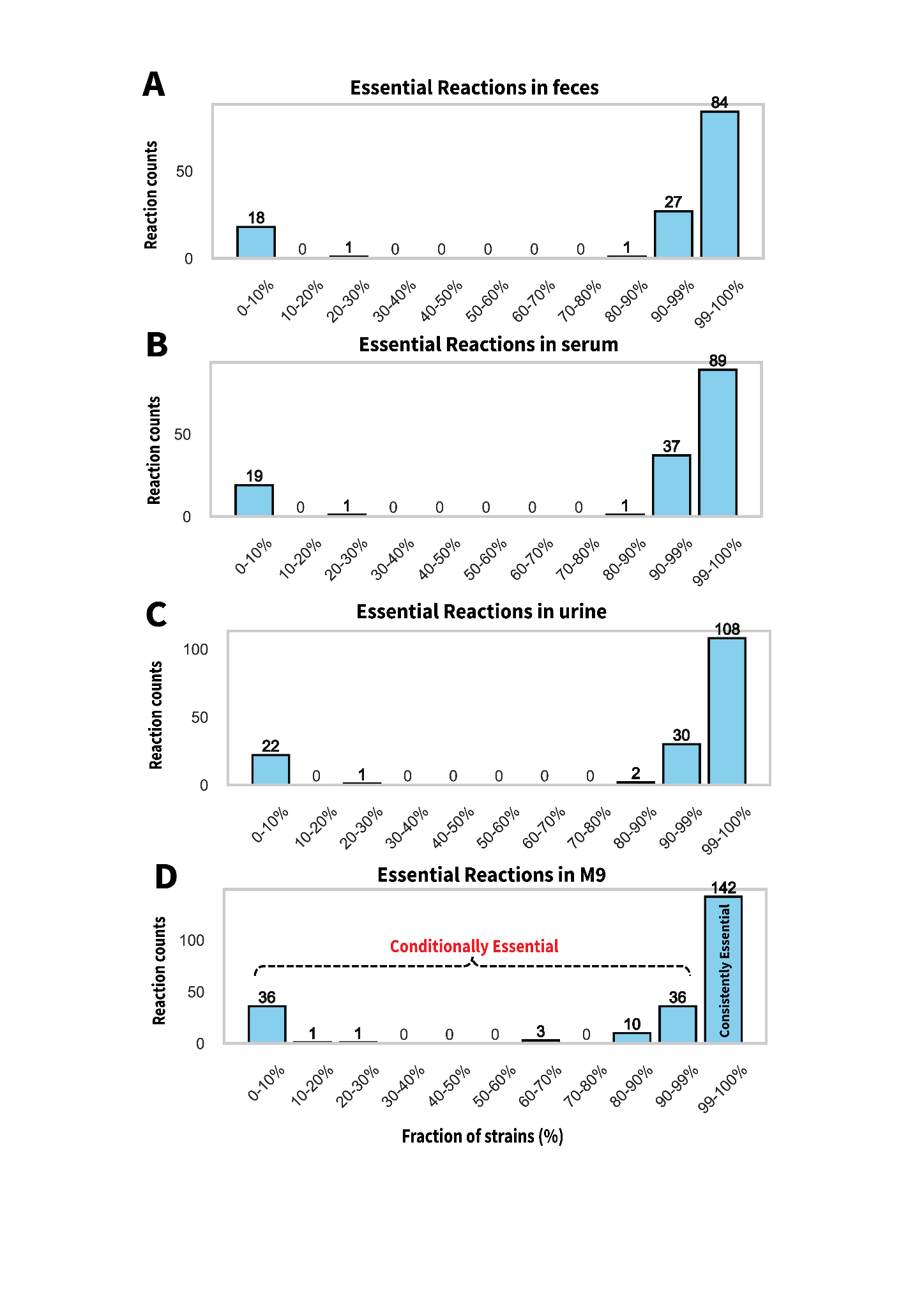
***

***S4 Fig.*** *Essential reactions across different media, barplot represents count of essential reactions grouped by frequency of occurrence across panGEM in different media. Frequency groups are presented on X axis, Y axis depicts distinct reactions count. A) Essential reactions in feces. B) Essential reactions in serum. C) Essential reactions in urine. D) Essential reactions in M9.*
