# Supplementary material for: Rare metabolic gene essentiality is a determinant of microniche adaptation in Eschherichia coli
Source: PLoS Pathog. 2025 Dec 8;21(12):e1013775. doi: 10.1371/journal.ppat.1013775 (PMC12704874; doi:10.1371/journal.ppat.1013775)
Supplement: S5 Fig — (DOCX) [file ppat.1013775.s005.docx]

*
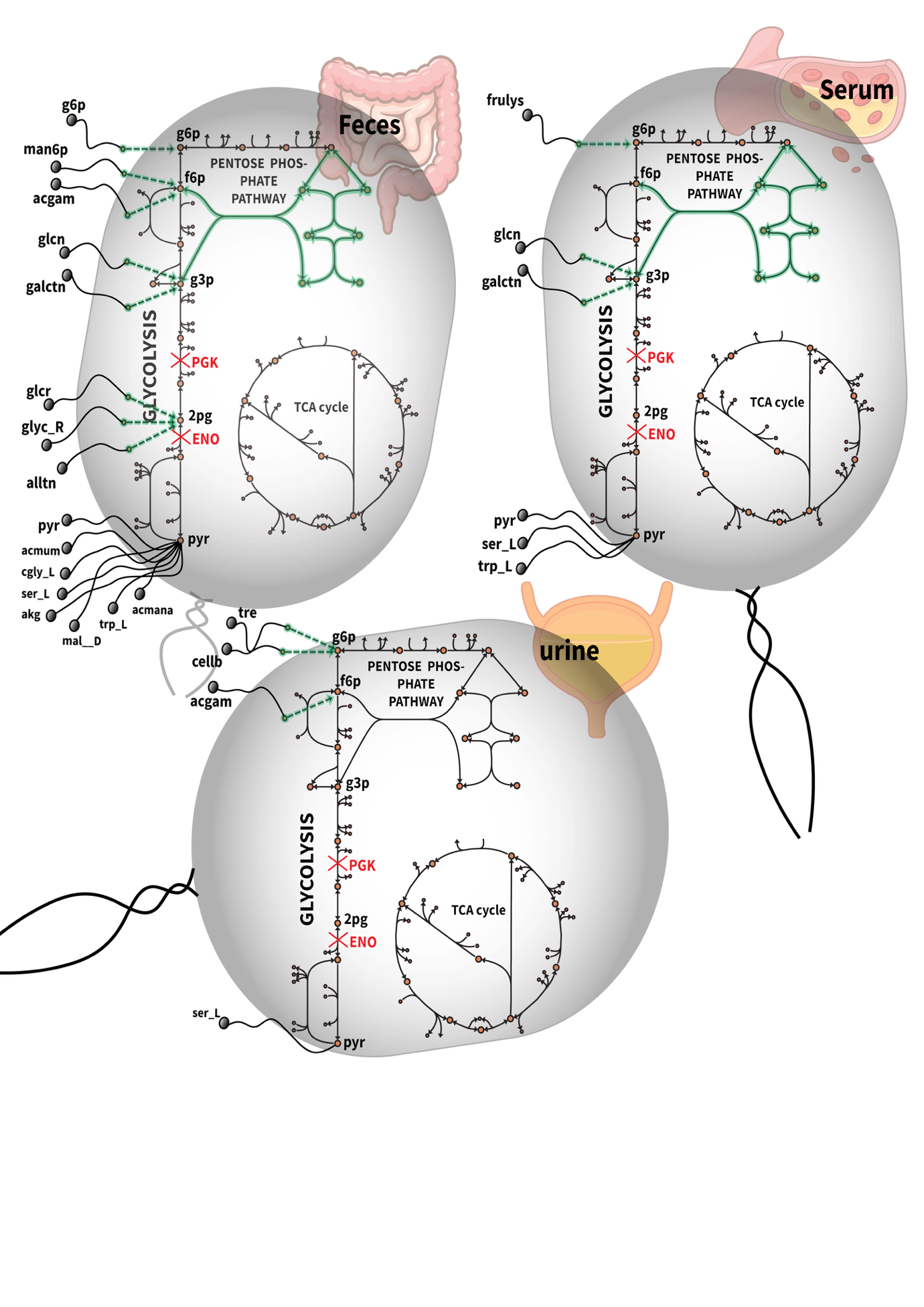
*

***S5 Fig: Urine-Specific Essential Reactions.*** *Illustration of two urine-specific essential reactions linked to available nutrients in urine media. Nutrients feeding glycolysis are shown outside the bacterium, while the glycolysis pathway inside shows essentiality of PGK and ENO in urine, but not in feces or serum. Green shading indicates predicted non-zero flux in the metabolic pathways.*
