# Supplementary material for: Rare metabolic gene essentiality is a determinant of microniche adaptation in Eschherichia coli
Source: PLoS Pathog. 2025 Dec 8;21(12):e1013775. doi: 10.1371/journal.ppat.1013775 (PMC12704874; doi:10.1371/journal.ppat.1013775)
Supplement: S1 Text — (DOCX) [file ppat.1013775.s011.docx]

**S1 Text. Feces Environment-Specific Uptake Profile**. (FVA) predicted that *E. coli* strains could utilize up to 163 compounds in feces, with 62 compounds unique to this environment (Figure 2A). pFBA further indicated that *E. coli* JJ1887 can utilize 119 compounds in feces, including 41 exclusive to this environment (Figure 2C). The primary nutrient categories available in feces included carbohydrates and sugar derivatives (41 compounds), nucleotides and derivatives (21 compounds), amino acids and derivatives (20 compounds), ions and inorganic compounds (14 compounds), organic acids (10 compounds), vitamins and cofactors (5 compounds), glycerophospholipids (4 compounds), and miscellaneous metabolites (5 compounds).

pFBA further predicted that the primary end-products of glycolysis in feces are formate (59.1 mmol/gDCW/h), acetate (41.9 mmol/gDCW/h), and ethanol (22.8 mmol/gDCW/h), characteristic of anaerobic metabolism. The electron transport chain remains inactive due to anaerobic conditions (Figure 2C).
