# Supplementary material for: Rare metabolic gene essentiality is a determinant of microniche adaptation in Eschherichia coli
Source: PLoS Pathog. 2025 Dec 8;21(12):e1013775. doi: 10.1371/journal.ppat.1013775 (PMC12704874; doi:10.1371/journal.ppat.1013775)
Supplement: S2 Text — (DOCX) [file ppat.1013775.s012.docx]

**S2 Text. Serum Environment-Specific Uptake Profile.** In serum, FVA identified 151 compounds that *E. coli* strains can utilize, with 27 specific to this environment (Figure 2A). pFBA analysis further predicted that *E. coli* JJ1887 could take up 76 metabolites in serum, including amino acids and derivatives (12), nucleotides and derivatives (14), carbohydrates and sugar derivatives (18), ions and inorganic compounds (18), organic acids (3), and miscellaneous metabolites (5). Among these, nine serum-specific metabolites were consumed (Figure 2C).

Glycolytic flux in serum results in lactate as the primary end product (21.3 mmol/gDCW/h), along with acetate (5.44 mmol/gDCW/h) and CO2 (10.5 mmol/gDCW/h). The presence of oxygen in serum (6960.0 ± 410.0 µM) [22] supports an active electron transport chain, further distinguishing this metabolic state from that in other colonization sites (Figure 2C).
