# Supplementary material for: Rare metabolic gene essentiality is a determinant of microniche adaptation in Eschherichia coli
Source: PLoS Pathog. 2025 Dec 8;21(12):e1013775. doi: 10.1371/journal.ppat.1013775 (PMC12704874; doi:10.1371/journal.ppat.1013775)
Supplement: S3 Text — (DOCX) [file ppat.1013775.s013.docx]

**S3 Text. Urine Environment-Specific Uptake Profile.** FVA predicted that *E. coli* strains could utilize 105 compounds in urine, with five unique to this environment (Figure 2A). pFBA analysis further indicated that *E. coli* JJ1887 is capable of taking up 60 metabolites in urine, spanning amino acids and derivatives (13), nucleotides and derivatives (12), carbohydrates and sugar derivatives (12), ions and inorganic compounds (14), organic acids (2), and miscellaneous metabolites (4). Among these, only one urine-specific metabolite, D-cellobiose, was predicted to be consumed (Figure 2C).

Distinct from other colonization sites, the primary glycolytic end-products in feces are formate, acetate, and ethanol, with CO_2_ being consumed rather than produced, and an inactive electron transport chain due to anaerobic conditions. Collectively, these findings illustrate a metabolic shift from a highly catabolic state in feces to a predominantly anabolic state in urine, characterized by lower catabolic pressure.
