# Supplementary material for: Rare metabolic gene essentiality is a determinant of microniche adaptation in Eschherichia coli
Source: PLoS Pathog. 2025 Dec 8;21(12):e1013775. doi: 10.1371/journal.ppat.1013775 (PMC12704874; doi:10.1371/journal.ppat.1013775)
Supplement: S4 Text — (DOCX) [file ppat.1013775.s014.docx]

**S4 Text.** **Media-Specific Reaction Essentiality Determined by Nutrient Availability Rather Than Genetic Variation.** By comparing essential reactions across simulated media, regardless of their consistency across strains or media, we identified a total set of 234 essential reactions predicted to occur across all four media. Additionally, media-specific essential reactions—those essential in only one simulated medium—and reactions essential in overlapping combinations of two or three media were identified. Notably, all 71 M9-specific essential reactions were essential across all strains. Serum and feces did not yield any exclusive essential reactions whereas knockout simulations in urine revealed three urine-specific essential reactions, all within the glycolysis pathway. Among these, Glyceraldehyde-3-phosphate dehydrogenase (GAPD) was predicted to be essential in 9.68% of strains, respectively. In contrast, Enolase (ENO) and Phosphoglycerate kinase (PGK) were consistently essential across all strains, showing 100% essentiality in strains growing in urine.

To further examine the basis of media-specific essentiality, pFBA was conducted to simulate the impact of different media on ENO and PGK knockouts. The predictions indicated that each medium provides unique nutrients linking to specific stages of glycolysis. pFBA predicted that supplementing the urine medium with either glcn or galctn could restore growth in ENO and PGK knockout strains, highlighting the role of nutrient availability in determining essentiality (Supplementary Figure 5).
